# Challenges and Insights into integrating Single-Particle Cryo-EM and MicroED

**DOI:** 10.1063/4.0001084

**Published:** 2025-10-27

**Authors:** Ampon C Saeher, Tamir Gonen

**Affiliations:** UCLA, Los Angeles, CA, USA

## Abstract

The adaptation of single-particle cryo-electron microscopy (cryo-EM) on a cryo-EM platform primarily designed for microcrystal electron diffraction (MicroED) presents unique challenges and opportunities. Our efforts to expand the capabilities of a MicroED-focused system to accommodate single-particle workflows have yielded valuable insights that will be of interest to the structural biology community. Key hurdles included improving sample preparation for high-resolution data collection, optimizing high throughput data collection, and refining data processing pipelines to obtain high resolution structures. Additionally, we explored the integration of focused ion beam (FIB) milling to enhance sample quality and reproducibility, addressing some of the inherent challenges associated with vitrified sample preparation on samples that are too thick for electron diffraction. Here, we share our experiences, highlight critical obstacles, and discuss potential strategies for combining the workflow single-particle cryo-EM implementation and MicroED. Our findings contribute to ongoing efforts to broaden access to cryo-EM methodologies and expand their applicability across diverse research environments for structural biology studies.

